# Development and Validation of a Novel Method for Converting the Japan Coma Scale to Glasgow Coma Scale

**DOI:** 10.2188/jea.JE20220147

**Published:** 2023-10-05

**Authors:** Mikio Nakajima, Yohei Okada, Tomohiro Sonoo, Tadahiro Goto

**Affiliations:** 1 Emergency Life-Saving Technique Academy of Tokyo, Foundation for Ambulance Service Development, Tokyo, Japan; 2 Department of Clinical Epidemiology and Health Economics, School of Public Health, The University of Tokyo, Tokyo, Japan; 3 TXP Medical Co. Ltd., Tokyo, Japan; 4 Department of Primary Care and Emergency Medicine, Graduate School of Medicine, Kyoto University, Kyoto, Japan

**Keywords:** Glasgow Coma Scale, Japan Coma Scale, conversion, concordance, validation

## Abstract

**Background:**

The Japan Coma Scale (JCS) is the most frequently adopted method for evaluating level of consciousness in Japan. However, no validated method for converting the JCS to the Glasgow Coma Scale (GCS) exists. The aims of the present study were to develop and validate a method to convert the JCS to GCS.

**Methods:**

This is a multicenter retrospective analysis involving three emergency departments (EDs) in Japan. We included all adult patients who visited the ED between 2017 and 2020. The participating facilities were divided into two cohorts—one cohort to develop a table to convert the JCS to GCS (development cohort), and the other cohort to validate the conversion table (validation cohort). The conversion table of the JCS to GCS was developed based on the median values of the GCS. The outcome was the concordance rate between the JCS and GCS.

**Results:**

We identified 8,194 eligible patients. The development cohort included 7,373 patients and the validation cohort included 821 patients. In the validation cohort, the absolute and relative concordance rates were 80.3% (95% confidence interval, 77.4–82.9%) and 93.2% (95% confidence interval, 91.2–94.8%), respectively.

**Conclusion:**

This study developed and validated a novel method for converting the JCS to GCS. Assuming the offset by a single category between the JCS and GCS is acceptable, the concordance rate was over 90% in the general adult patient population visiting the ED. The conversion method may assist researchers to convert JCS scores into GCS scores, which are more commonly recognized among global audiences.

## INTRODUCTION

An objective assessment of level of consciousness is critical in the emergency department (ED). The Glasgow Coma Scale (GCS), first introduced in 1974, has been widely adopted internationally and has become the de facto gold standard for evaluating conscious level.^1^ On the other hand, the Japan Coma Scale (JCS) is the most commonly applied method for assessing patients’ consciousness level in Japan given its simplicity and applicability.^2^^–^^4^ The JCS consists of four main grades of consciousness based on reactive eye-opening, which can be summarized as follows: 0, alert consciousness; 1–3 (single-digit), awake without any stimuli; 10–30 (double-digits), arousable by some stimuli but reverts to previous state if stimulus stops; and 100–300 (triple-digits), unarousable by any stimuli (Table 1).^3^ Based on these grades, the JCS has 10 categories of scores ranging from 0 to 300. In contrast, the GCS has 13 categories of scores.

**Table 1.** Japan Coma Scale scoring

		Level of consciousness
	0	Alert
Single-digit	Awake without any stimuli	
	1	Almost fully conscious but not normal
	2	Unable to recognize time, place, and person
	3	Unable to recall name or date of birth
Double-digits	Arousable by some stimuli but reverts to previous state if stimulus stops	
	10	Arousable by being spoken to
	20	Arousable by loud voice
	30	Arousable only by repeated mechanical stimuli
Triple-digits	Unarousable by any forceful stimuli	
	100	Unarousable but responds to avoid the stimuli
	200	Unarousable but responds with slight movements, including decerebrate or decorticate postures
	300	Does not respond at all

The JCS is adopted as a standard prehospital criterion for hospital selection and is widely used by hospital and emergency medical technicians throughout Japan. In addition to this, large-scale Japanese medical databases, including nationwide administrative claims and discharge data, often contain the JCS scores as an indicator of consciousness level instead of the GCS.^5^^–^^7^ However, there are no validated methods for converting JCS to GCS scores. Due to the lack of a validated conversion method, the findings reported in clinical research conducted in Japan are often discounted overseas. Devising a JCS-GCS conversion method is a critical aspect of bridging the divide between these two systems, which could lead to the progress and development of research in the field of emergency medicine.

The aims of the present study are (i) to demonstrate the relationship between the JCS and GCS, (ii) establish a method for converting the JCS to GCS, and (iii) evaluate the validity of the conversion method using actual cases documented in the general ED setting.

## METHODS

### Design and settings

The present study is a multicenter retrospective analysis involving three EDs in Japan between January 2017 and October 2020. The actual periods of data collection differed between facilities. Participating institutions included Hitachi General Hospital (data collection periods, January 2017 to October 2020), Saiseikai Utsunomiya Hospital (April 2020 to September 2020), and the Japanese Red Cross Society Kyoto Daiichi Hospital (April 2020 to October 2020). All participating healthcare facilities are tertiary-care emergency hospitals and board specialist training centers certified by the Ministry of Health, Labour and Welfare of Japan and the Japanese Association for Acute Medicine, respectively. There are approximately 13,000–15,000 ED visits and 5,000–6,000 patients transported via emergency medical services annually for each participating facility.^8^

### Data source and collection

In these hospitals, the medical charts are maintained using the NEXT Stage ER system (TXP Medical Co., Ltd. Tokyo, Japan), which supports healthcare professionals record clinical information, such as chief complaints, history of present illness, past medical history, medications used, physical assessments, vital signs, and clinical diagnoses as structured data. The clinical diagnoses were recorded with free-text and the information was automatically encoded to the International Statistical Classification of Diseases, Tenth Revision (ICD-10) codes. Clinical diagnoses in the database have been previously validated.^9^ Several clinical studies using the database have been previously published.^8^^,^^10^^,^^11^

### Patient selection

We identified consecutive data on patients who visited the above EDs during the study period. Patients aged ≤16 years and those missing either the JCS or GCS on ED arrival were excluded from the present study.

### Variables

Patient characteristics included age, sex, emergency medical service use, level of consciousness at the timing of ED arrival, physician’s primary diagnosis at the ED, and disposition of the ED (admission or discharge). The level of consciousness was recorded using the 10 JCS categories and 13 GCS categories (scores ranging 3–15). The scores were evaluated and recorded by the emergency physicians or nurses upon patient arrival at the ED. The physician’s diagnosis at the time of ED discharge was divided into the following 17 categories based on the first letter of the ICD-10 code: infectious and parasitic diseases (ICD-10 codes, A and B); neoplasms (C and D); endocrine, nutritional and metabolic diseases (E); mental and behavioral disorders (F); diseases of the nervous system (G); diseases of the eye, adnexa, ear, and mastoid process (H); diseases of the circulatory system (I); diseases of the respiratory system (J); diseases of the digestive system (K); diseases of the skin and subcutaneous tissue (L); diseases of the musculoskeletal system and connective tissue (M); diseases of the genitourinary system (N); pregnancy, childbirth, puerperium, and perinatal condition (O and P); symptoms, signs, and abnormal clinical and laboratory findings, not elsewhere classified (R); injury, poisoning, and certain other consequences of external causes (S and T); others (X, Z and Q); and missing data.

### Development and validation cohorts

The participating facilities were divided into two cohorts: i) development cohort (Hitachi General Hospital) used to develop the table to convert the JCS to GCS, and ii) validation cohort (Saiseikai Utsunomiya Hospital and the Japanese Red Cross Society Kyoto Daiichi Hospital) used to validate and assess the concordance rate. The outcome of the present study was the concordance rate between the JCS and GCS.

### Statistical analysis

To describe patient characteristics in the development and validation cohorts, continuous variables were reported as median and interquartile range (IQR) and categorical variables were reported as count and percentage.

In the development cohort, we described the median value of the GCS corresponding to each JCS score. Then, we developed a table to convert the JCS to GCS based on the median values.

For external validation, the developed conversion table was applied to the validation cohort and the concordance rates based on the table conversions were described. “Absolute concordance” was defined as the percentage of exact concordance with the developed conversion table and “relative concordance” was defined as the percentage of concordance which allows for offset by a single GCS point (+/− 1).

We calculated the proportion of outcomes and the 95% confidence interval (CI) using the Clopper–Pearson interval. Stata/BE 17 (Stata Corp, College Station, TX, USA) and R version 4.1.2 (The R Foundation for Statistical Computing, Vienna, Austria) were used for statistical analysis.

### Sensitivity analysis

Because the percentage of patients with alert consciousness influence the outcome (ie, concordance rate), we performed a sensitivity analysis excluding patients with alert consciousness (JCS = 0). The outcomes of the sensitivity analysis were the same as the primary analysis (absolute and relative concordance).

### Ethics

The study protocol was approved by the Ethics Committee of TXP Medical Co., Ltd (approval number, TXPREC-002). The requirements for informed consent were waved due to the retrospective nature of the study. Only anonymized data was collected from each participating institution.

## RESULTS

### Study participants

**Figure 1.**
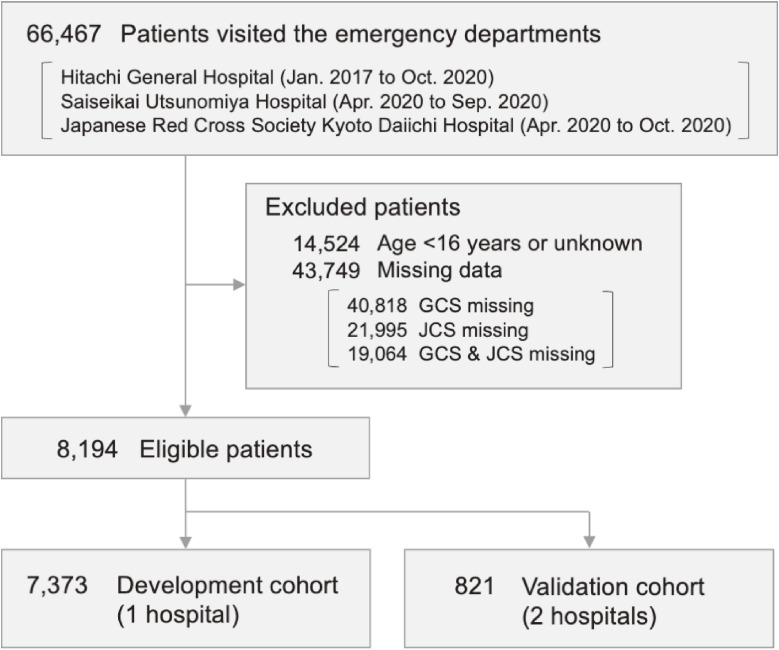
Study flowchart. JCS, Japan Coma Scale; GCS, Glasgow Coma Scale.

After application of the inclusion and exclusion criteria, we identified 8,194 eligible patients during the study period (Figure 1). The development cohort included 7,373 patients from one hospital, and the validation cohort included 821 patients from the remaining two hospitals. Table 2 demonstrates the baseline characteristics of patients who visited the EDs in the development and validation cohorts. The median age in the development and validation cohorts were 74 (IQR, 58–82) and 70 (IQR, 50–81) years, respectively. The percentages of emergency medical service use were 81.3% and 66.3%, respectively. Approximately half of the patients were admitted to the hospital in both cohorts. Circulatory disease, injury, digestive disease and respiratory disease were the most frequent diagnoses in both cohorts. JCS and GCS in each cohort is shown in eTable 1.

**Table 2.** Patient characteristics in the development and validation cohorts

Variables	Development cohort (*n* = 7,373)		Validation cohort (*n* = 821)	
Age, years, median (IQR)	74	(58–82)	70	(50–81)
Age category, years				
≤34	612	(8.3)	94	(11.4)
35–54	1,001	(13.6)	155	(18.9)
55–64	773	(10.5)	83	(10.1)
65–84	3,540	(48.0)	380	(46.3)
≥85	1,424	(19.3)	107	(13.0)
Sex, male	4,078	(55.4)	434	(53.0)
Emergency medical service use	5,836	(81.3)	509	(66.3)
Admission	3,658	(49.6)	399	(48.6)
Diagnosis at emergency department [first letter of the ICD-10 codes]				
Infectious and parasitic diseases [A, B]	241	(3.3)	13	(1.6)
Neoplasms [C, D]	209	(2.8)	13	(1.6)
Endocrine, nutritional and metabolic diseases [E]	266	(3.6)	36	(4.4)
Mental and behavioral disorders [F]	167	(2.3)	21	(2.6)
Diseases of the nervous system [G]	362	(4.9)	28	(3.4)
Diseases of the eye, adnexa, ear and mastoid process [H]	144	(2.0)	12	(1.5)
Diseases of the circulatory system [I]	1,472	(20.0)	115	(14.0)
Diseases of the respiratory system [J]	600	(8.1)	48	(5.8)
Diseases of the digestive system [K]	633	(8.6)	82	(10.0)
Diseases of the skin and subcutaneous tissue [L]	78	(1.1)	16	(1.9)
Diseases of the musculoskeletal system and connective tissue [M]	175	(2.4)	19	(2.3)
Diseases of the genitourinary system [N]	316	(4.3)	30	(3.7)
Pregnancy, childbirth, puerperium and perinatal condition [O, P]	5	(0.1)	3	(0.4)
Symptoms, signs and abnormal clinical and laboratory findings [R]	870	(11.8)	135	(16.4)
Injury, poisoning and other consequences of external causes [S, T]	1,231	(16.7)	135	(16.4)
Others [X, Z, Q]	18	(0.2)	2	(0.2)
Missing data	586	(7.9)	113	(13.8)

ICD-10, International Statistical Classification of Diseases, Tenth Revision; IQR, interquartile range.

Data are shown as *n* (%) unless otherwise specified.

### Development and validation of the conversion table

**Figure 2.**
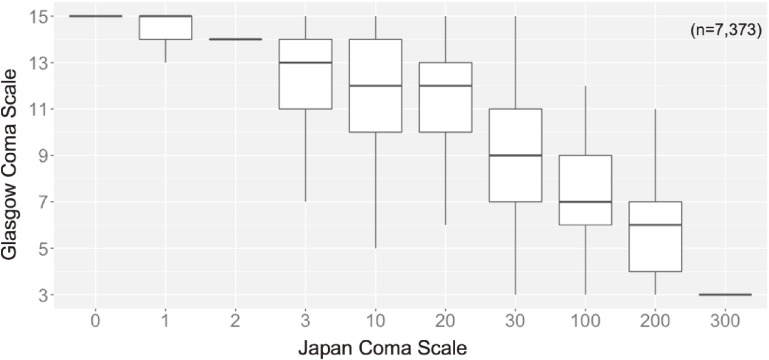
Relationship between the Japan Coma Scale and Glasgow Coma Scale in the development cohort. Data are presented as box plots. Lines in the box represent median values and box edges represent 25th to 75th percentiles.

Figure 2 demonstrates the relationship between the JCS and GCS in the development cohort. Based on the median value of the GCS for each JCS, we created a table to convert the JCS to GCS (Table 3). Using this table, if a patient in the validation cohort had a JCS of 30, absolute concordance would be a GCS score of 9. Relative concordance using the same example would be a GCS ranging between 8 and 10. In the validation cohort, absolute concordance was 80.3% (95% CI, 77.4–82.9%) and relative concordance was 93.2% (95% CI, 91.2–94.8%). In the sensitivity analysis excluding patients with alert consciousness (JCS = 0), absolute concordance was 46.1% (95% CI, 40.0–52.2%) and relative concordance was 80.5% (95% CI, 75.3–85.1%) (Table 4).

**Table 3.** Developed conversion table of the Japan Coma Scale to the Glasgow Coma Scale

Japan Coma Scale	Glasgow Coma Scale
0	15
1	15
2	14
3	13
10	12
20	12
30	9
100	7
200	6
300	3

**Table 4.** Outcomes in the validation cohort

Outcomes	Percentage	(95% CI)
Primary analysis (*n* = 821)		
Absolute concordance^a^	80.3%	(77.4–82.9%)
Relative concordance^b^	93.2%	(91.2–94.8%)
Sensitivity analysis (*n* = 267)^c^		
Absolute concordance^a^	46.1%	(40.0–52.2%)
Relative concordance^a^	80.5%	(75.3–85.1%)

CI, confidence interval.

^a^Absolute concordance was defined as the percentage of exact concordance with the developed conversion table.

^b^Relative concordance was defined as the percentage of concordance which allows for offset by a single category with the developed conversion table.

^c^Sensitivity analysis was performed omitting patients with alert consciousness (Japan Coma Scale = 0).

## DISCUSSION

In the present study, we established a method to covert the JCS to GCS for use in general ED indications using a multicenter ED database in Japan. Additionally, we evaluated the validity of the conversion method using the concordance rate between the JCS and GCS. Absolute concordance was over 80% and the relative concordance exceeded 90%. Although absolute concordance in patients without alert consciousness was below 50%, the relative concordance rate was preserved at over 80%.

The JCS is widely used not only in clinical practice, but also in countless clinical studies in Japan.^5^^–^^7^ Previous studies have demonstrated that the JCS level was beneficial in predicting in-hospital mortality in trauma patients.^3^^,^^4^ Another study revealed that the JCS was associated with activity of daily living at 30 days after stroke and mortality in stroke patients.^2^ The JCS is a formidable coma scale; however, the JCS is exclusively utilized in Japan, which is one of the factors impeding the direct application of Japanese clinical study outcomes to overseas practice. The present study is uniquely designed to help translate the JCS into a more broadly adopted consciousness scoring system—the GCS—in order to facilitate dissemination of clinical studies utilizing Japanese databases to a global audience.

The sum of the GCS score is used in clinical settings as well as several classification systems in the intensive care unit, such as the Acute Physiologic and Chronic Health Evaluation (APACHE), the Sepsis-related Organ Failure Assessment (SOFA), and the Simplified Acute Physiology Score (SAPS).^12^^–^^14^ When the level of consciousness is assessed only by the JCS—and GCS records are absent—these classification systems cannot be retrospectively calculated. The JCS and GCS are not seamlessly convertible because of the inherent differences between the components evaluated. To the best of our knowledge, there are no validated methods to convert the JCS to GCS. The present study established and validated a novel method for converting the JCS to GCS. Assuming the offset by a single category between the JCS and GCS is acceptable, the concordance rate was over 90%. The established conversion method in the present study can be broadly utilized by clinical studies using real world big data.

One of the strengths of this study is its multi-centered nature, which allowed for enhanced validity. Additionally, we included all emergency patients—not just trauma or stroke—which ensures the broader applicability of the conversion method described in this study.

There are several potential limitations that should be acknowledged. First, the medical staff who evaluated and recorded the JCS and GCS at the time of ED arrival were not recorded in detail. Occupation (medical doctor or nurse), years of experience and specialty may influence the reliability and concordance of the JCS and GCS.^15^ Second, we developed a method to convert the JCS to GCS based on the median value of the GCS; however, the number of cases involving a JCS of 3–200 was limited and the IQRs of the range varied. These levels of intermediate consciousness may lead to a poor concordance rate between the JCS and GCS. Because the JCS and GCS are not continuous variables, we did not apply a regression analysis in the development of a clinically convenient but highly functional conversion table. Third, the outcomes in the present study (ie, concordance rate) could have been influenced by the proportion of patients with alert consciousness who may have higher concordance with their respective scores (JCS = 0 and GCS = 15). To address this issue, we conducted a sensitivity analysis excluding patients with alert consciousness (JCS = 0). Fourth, among 66,467 ED visits, 43,749 (66%) patients were excluded due to missing JCS or GCS values. This may have influenced the outcomes and inadvertently introduced a selection bias; however, it can be assumed that level of consciousness was recorded using either the JCS or GCS (and not both) because most patients visiting the ED are generally alert and oriented (eg, acute respiratory tract infections). Patients who were evaluated using both the JCS and GCS may have presented with unique factors (eg, when the patient’s state of consciousness was difficult to assess via only a single coma scale). Finally, the JCS and GCS should be assessed independently because the components utilized for evaluation differs. The conversion table established in this study may not ensure absolute conversion of the JCS to GCS in individual patients; however, it has displayed promising results with regard to the patient population examined in this study.

In conclusion, this multicenter study generated a novel method to convert the JCS to GCS and validated concordance between the JCS and GCS. Assuming the offset by a single category between the JCS and GCS is acceptable, the concordance rate was over 90% in the general adult patient population visiting the ED. The conversion method described in the present study may assist Japanese researchers to translate JCS into the GCS format which is more commonly recognized among global audiences.

## SUPPLEMENTARY MATERIAL

eTable 1. Consciousness level in the development and validation cohorts
