# Supplementary material for: Development and Validation of a Novel Method for Converting the Japan Coma Scale to Glasgow Coma Scale
Source: J Epidemiol. 2023 Oct 5;33(10):531–5. doi: 10.2188/jea.JE20220147 (PMC10483104; doi:10.2188/jea.JE20220147)
Supplement: Supplementary file 1 [file je-33-531-s001.pdf]

eTable 1. Consciousness level in the development and validation cohorts

	Development cohort (n=7,373)	Validation cohort (n=821)
Japan Coma Scale		
0	4,784 (64.9)	554 (67.5)
1	808 (11.0)	82 (10.0)
2	352 (4.8)	73 (8.9)
3	428 (5.8)	40 (4.9)
10	239 (3.2)	33 (4.0)
20	82 (1.1)	5 (0.6)
30	72 (1.0)	2 (0.2)
100	109 (1.5)	8 (1.0)
200	124 (1.7)	14 (1.7)
300	375 (5.1)	10 (1.2)
Glasgow Coma Scale		
3	337 (4.6)	8 (1.0)
4	33 (0.4)	2 (0.2)
5	29 (0.4)	2 (0.2)
6	107 (1.5)	8 (1.0)
7	89 (1.2)	10 (1.2)
8	61 (0.8)	7 (0.9)
9	80 (1.1)	6 (0.7)
10	109 (1.5)	8 (1.0)
11	131 (1.8)	16 (1.9)
12	143 (1.9)	8 (1.0)
13	293 (4.0)	25 (3.0)
14	878 (11.9)	123 (15.0)
15	5,083 (68.9)	598 (72.8)

Data are shown as n (%).
